# Seismic Performance of RC Frame Structures with Masonry Infill Walls Strengthened by Mortar Surface Layers

**DOI:** 10.3390/ma19214531

**Published:** 2026-10-27

**Authors:** Chen Qiu, Tiehua Shi, Yawei Gao, Ying Huang, Guang Yang, Yanhua Zhang, Yuyao Wu, Yingmu Jiang

**Affiliations:** 1 Department of Building Materials, China Academy of Building Research, Beijing 100013, China; 19115038@bjtu.edu.cn (C.Q.);; 2 Disaster Prevention Research Center, Ministry of Housing and Urban-Rural Development, Beijing 100013, China; 3 Beijing Institute of Architectural Design Co., Ltd., Beijing 100045, China

**Keywords:** masonry infill wall, frame structure, seismic performance, mortar surface reinforcement

## Abstract

Although infill masonry walls in frame structures are classified as non-structural components, they can still resist a portion of seismic forces during earthquakes. Historical earthquake damage indicates that infill masonry walls in frame structures often act as the first seismic defense line and fail first, thereby mitigating or delaying damage to the main frame to a certain extent. Such damage cases are frequently observed. Therefore, it is necessary to investigate the enhancement of the seismic performance of the main structure contributed by infill masonry walls, and further to study the effect of infill-wall retrofitting on the seismic behavior of frame structures. The mortar surface reinforcement method, owing to its convenient construction and low cost, is one of the commonly used techniques for enhancing the seismic performance of masonry walls. This paper conducts finite element analysis on a frame structure located in a seismic fortification zone of intensity 8. The seismic performance of a bare frame, a frame with infill masonry walls, and frames with mortar surface reinforcement applied to infill walls at different locations are compared. Parameters including inter-story drift ratio, floor seismic forces, column shear forces, and load–displacement responses under various earthquake actions are analyzed. The results demonstrate that, for the numerical model employing an idealized interface assumption, the presence of infill walls significantly reduces the inter-story drift and column shear forces of the frame structure. Compared with the unretrofitted model, the seismic bearing capacity of the frame structure with mortar-reinforced infill walls increases by 18.57–36.19%. Among the different retrofitting schemes, the case with vertical and horizontal reinforcement applied to half of the wall area each achieves similar inter-story drift control as other schemes, while simultaneously reducing the floor seismic forces and the shear forces carried by frame columns. Thus, partial reinforcement of infill walls is recommended in structural retrofitting design, accompanied by experimental validation to quantitatively assess the retrofitting efficacy whenever possible. The findings of this study can serve as a reference for improving the seismic bearing capacity of frame structures through the mortar surface reinforcement method applied to infill masonry walls.

## 1. Introduction

Reinforced concrete (RC) frame structures are load-bearing systems composed of rigidly connected reinforced concrete beams and columns. Infill walls, as non-load-bearing components, primarily serve the functions of enclosure and space division. From a mechanical perspective, RC frame structures formed by beams and columns are skeletal structures. Even when the composite action between floor slabs and beams is considered, their horizontal lateral stiffness is smaller than that of structures with infill walls under the condition of identical member dimensions. Consequently, under strong earthquake actions, RC frame structures are prone to large horizontal displacements, and the entire structure is highly vulnerable to severe damage [1]. Although infill walls are non-structural components, they are usually embedded in concrete or reinforced with tie bars during construction to strengthen the connection with surrounding frame columns and beams. This practice indicates that infill walls participate in the overall force-resisting behavior of the structure [2,3]. A large number of experiments and post-earthquake investigations have also revealed that during earthquakes, infill walls act as the first seismic defense line, absorbing a portion of the seismic forces and thus affecting the seismic performance of RC frame structures [4], which similarly confirms that infill walls participate in the overall force-bearing of the frame structure.

To explicitly define the influence of infill walls on the seismic performance of reinforced concrete frame structures, researchers have performed extensive experimental studies and theoretical analyses. In the realm of experimental research, current studies primarily focus on flexible connection infill walls [5,6], various infill wall construction techniques [7], blast loads [8,9], boundary conditions [10], infill shear walls [11,12,13], precast slab infill walls [14,15], and infill walls with damped joint layers [16,17]. For theoretical analysis, researchers have examined the dynamic properties and seismic behavior of infill walls by considering factors such as period reduction coefficients [18], constitutive models [19,20,21], and semi-analytical methods [22]. Computational models have been developed based on experimental results to simulate observed phenomena [23,24], and design formulas have been proposed [25,26]. Post-earthquake damage investigations [4] have shown that brick or block infill walls in frame structures may sustain severe damage under seismic excitation, including horizontal cracks, diagonal cracks, “X”-shaped shear cracks, corner crushing, and out-of-plane collapse. Therefore, while infill walls are used to enhance the load-bearing capacity or stiffness of RC frames, appropriate strengthening measures must be implemented for existing infilled RC frames to prevent or delay seismic damage to the main structural system. Regarding the seismic retrofitting of existing RC frame structures, prior studies have investigated the effects of structural configuration [27] and stiffness distribution [28] on their global seismic performance.

The mortar surface layer strengthening method is widely applied in engineering practice due to its ease of construction, low cost, wide applicability, minimal disruption to the original building appearance, and limited impact on the existing structure [29,30]. Thus, this study adopts the mortar surface layer to strengthen existing masonry infill walls. Given that the research object of this paper is a complete structural system, experimental conditions are difficult to satisfy; previous experimental studies have mostly focused on single-story, single-span infilled frame systems [6,17]. In the numerical simulation, existing research on infill walls strengthened with mortar surface layers predominantly relies on separated refined models [31,32]. However, at the structural level, current studies lack sufficient simulation of the collaborative load-bearing behavior between the mortar surface layer and the masonry substrate. Therefore, it is necessary to develop an effective simplified numerical analysis model to explore the influence of mortar surface layer-strengthened masonry infill walls on the seismic performance of frame structures at the structural level. Existing macroscopic models for masonry walls typically utilize a single, homogeneous material layer with equivalent properties. In these approaches, surface reinforcement is either neglected or smeared into the global mechanical characteristics of the wall, thereby precluding the assignment of independent thickness, orientation, or constitutive behavior to the mortar overlay. To address this limitation, this study treats the mortar-reinforced masonry wall as a “composite material” by discretizing the cross-section along the thickness direction. This study uses a simplified numerical analysis method to establish an RC frame model incorporating mortar surface layer strengthening, aiming to investigate the strengthening effect of the mortar layer on RC frame structures and provide a reference for engineering applications.

## 2. Finite Element Model

### 2.1. Geometric Dimensions of RC Frame

**Figure 1:**
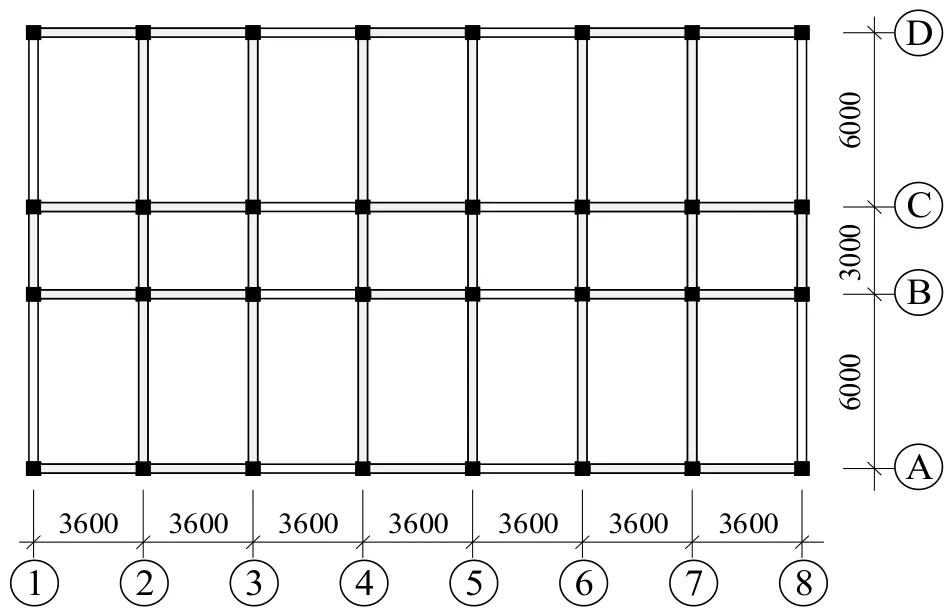
Layout diagram of the RC frame. (Unit: mm)

A five-story RC frame structure [23] situated in Seismic Intensity Zone 8 of China was selected as the research object. The structure consists of three transverse spans and seven longitudinal spans. The height of the first floor is 4 m, while the height of each subsequent floor is 3.6 m. The floor plan is illustrated in Figure 1. Infill walls are arranged along all transverse and longitudinal axes, with an identical number of walls deployed on each axis. Window openings are provided in the infill walls along longitudinal axes B and C: for Floors 2 to 5, the window openings measure 1.8 m × 1.8 m; for the first floor, the window openings are 1.8 m × 2.1 m. The height of the wall below the windows is 900 mm. Additionally, door openings with dimensions of 0.9 m × 2.0 m are installed in the infill walls along axes B and C.

The cross-sectional dimensions of the frame columns are 450 mm × 450 mm for Floors 1 and 2, and 400 mm × 400 mm for the remaining floors. The cross-sectional dimensions of the frame beams are 300 mm × 600 mm (transverse direction) and 300 mm × 450 mm (longitudinal direction). The thickness of the floor slab is 120 mm, and all masonry infill walls are uniformly 240 mm thick.

Reinforcement details of frame beams: Both the top and bottom reinforcements of the beams are 4Φ22; stirrups in the critical region are Φ8@200, with a confined zone length of 200 mm; stirrups in the non-confined region are Φ6@400. The floor slab is provided with double-layer reinforcement of Φ10@150. Reinforcement details of frame columns are presented in Table 1.

### 2.2. Material Properties

The concrete has a strength grade of C20, and the mortar surface layer is composed of M10 mortar. Both materials are modeled using the Concrete Damaged Plasticity (CDP) constitutive model implemented in ABAQUS v2025. The CDP model integrates plasticity and damage mechanics frameworks, employing isotropic damaged elasticity together with distinct, stress mode-dependent plastic flow rules for tension and compression—thereby capturing key nonlinear responses of concrete and mortar, including tensile cracking, compressive softening, and irreversible strain accumulation. The effective stress–strain relationship, accounting for progressive stiffness degradation due to damage, is given by:

*σ* = (1 − *d*)*E*_0_*ε* (1)

where *σ* denotes the stress, *d* is the scalar damage variable, *E*_0_ represents the initial elastic modulus of intact material, and *ε* is the total strain. Distinct tensile and compressive damage variables, denoted as *d*_t_ and *d*_c_ respectively, are introduced to independently characterize the evolving damage states under tension and compression.

The concrete uniaxial constitutive model specified in Appendix C of the Code for Design of Concrete Structures (GB 50010-2010) [33] is adopted. For uniaxial tension, the stress–strain curve is determined by the following equations:

*σ* = (1 − *d_t_*)*E_t_ε* (2)

(3) $$d_{t}=\left\{\begin{array}{c} 1-\rho_{t}\left[1.2-0.2x^{5}\right],x\leq 1\\ 1-\rho_{t}/\left[\alpha_{t}{\left(x-1\right)}^{1.7}+x\right],x>1 \end{array}\right.$$

*x* = *ε*/*ε_t_*_,*r*_ (4)

*ρ_t_* = *f_t_*_,*r*_/(*E_c_ε_t_*_,*r*_) (5)

where *α_t_* is the parameter governing the descending branch of the tensile stress-strain curve, taken according to Table C.2.3 of GB 50010-2010 [33]; *f_t,r_* is the representative value of the uniaxial tensile strength of concrete; and *ε_t,r_* is the peak tensile strain corresponding to *f_t,r_*. For uniaxial compression, the stress-strain curve is determined by the following equations:

*σ* = (1 − *d_c_*)*E_c_ε* (6)

(7) $$d_{c}=\left\{\begin{array}{c} 1-\rho_{c}n/(n-1+x^{n}),x\leq 1\\ 1-\rho_{c}/\left[\alpha_{c}{\left(x-1\right)}^{2}+x\right],x>1 \end{array}\right.$$

*x* = *ε*/*ε_c_*_,*r*_ (8)

*ρ_c_* = *f_c_*_,*r*_/(*E_c_ε_c_*_,*r*_) (9)

**Figure 2:**
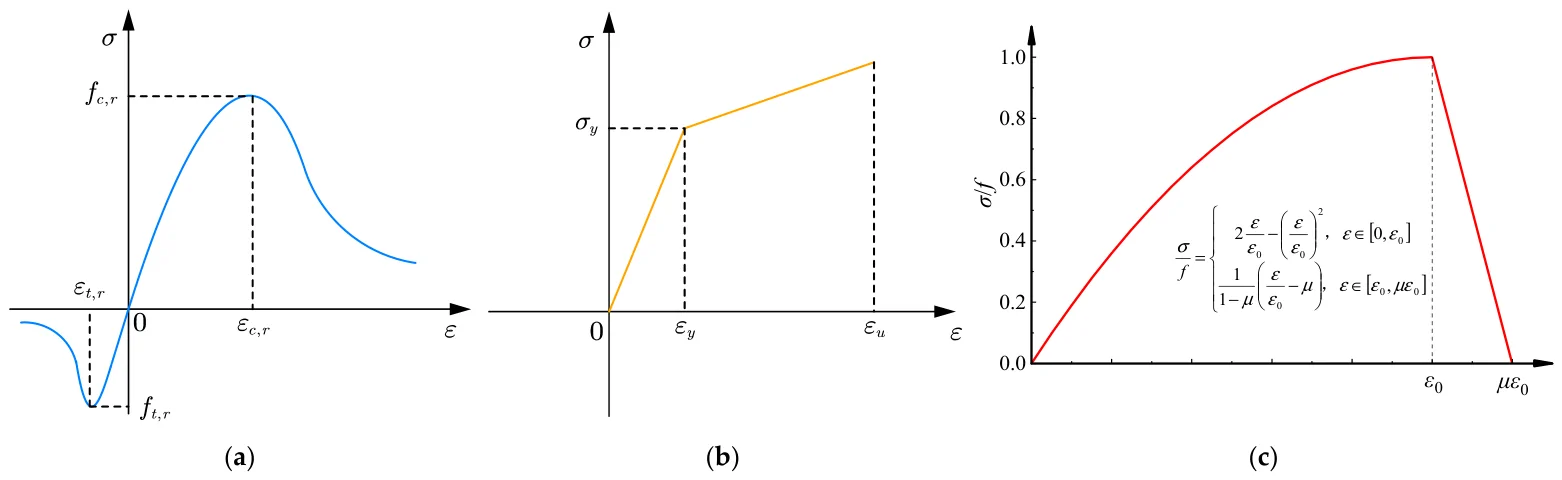
Stress–strain curves. (**a**) CDP model; (**b**) Linear hardening elastoplastic model; (**c**) Masonry infill wall.

*n* = *E_c_ε_c_*_,*r*_/(*E_c_ε_c_*_,*r*_ − *f_c_*_,*r*_) (10)

where *α_c_* is the parameter governing the descending branch of the compressive stress–strain curve, taken according to Table C.2.4 of GB 50010-2010; *f_c,r_* is the representative value of the uniaxial compressive strength of concrete; *ε*_c,r_ is the peak compressive strain corresponding to *f_c,r_*; and *n* is the shape parameter of the ascending branch. The corresponding tensile stress–strain curve is shown in Figure 2a. The elastic modulus, Poisson‘s ratio, and strength characteristics of C20 concrete are determined according to GB 50010-2010. The elastic modulus *E_c_* = 2.55 × 10^4^ MPa for C20 concrete is specified in Table 4.1.5 of GB 50010-2010. Clause 4.1.5 of GB 50010-2010 specifies a Poisson‘s ratio *ν*_c_ = 0.2 for concrete under elastic conditions. The uniaxial compressive strength *f_ck_* = 13.4 MPa and tensile strength *f_tk_* = 1.54 MPa for C20 are taken from the design values in clause 4.1.3 of GB 50010-2010. The characteristic cube strength *f_cu,k_* = 20 MPa was used as the basis for calculating the CDP constitutive parameters.

The damage evolution parameters in tension and compression are thus calculated based on the equivalent plastic strain. To fully define the plastic behavior in the CDP model, the following five plasticity parameters are specified: the dilation angle *ψ* is taken as 35°, the flow potential eccentricity *ε* equals 0.1, the ratio of initial equibiaxial compressive yield stress to initial uniaxial compressive yield stress *σ*_b0_/*σ*_c0_ is set to 1.16, the yield surface shape parameter *K_c_* is 0.667, and the viscosity parameter is assigned to 0.0001 to improve convergence of the cracking simulation algorithm.

Owing to the absence of corresponding experimental data of M10 Mortar, the empirical formula proposed in Ref. [34] is adopted. The elastic modulus of mortar can be expressed in terms of its compressive strength as *E* = 1057*f_c_*^0.84^ = 7313 MPa (where fc = 10.0 MPa). Given that M10 cement mortar exhibits physical properties analogous to those of concrete, its Poisson’s ratio is also taken as 0.2. The tensile strength of the mortar, *f_t_*, is adopted as 1.4 MPa.

The steel reinforcement is of grade HPB235, with its stress–strain relationship characterized by a linear hardening elastoplastic model, as shown in Figure 2b. The elastic modulus of HPB235 steel reinforcement is 2.1 × 10^5^ N/mm^2^. In this model, the stress–strain response is assumed to be linear elastic up to the yield stress *f_y_* = 235 MPa, followed by a linear hardening branch up to the ultimate tensile strength *f_u_* = 350 MPa.

The infill walls are constructed from MU7.5 solid clay bricks. The design value of the masonry compressive strength is *f* = 1.73 MPa. The ascending branch of the stress–strain curve follows a quadratic polynomial relationship, while the descending branch is linear, as illustrated in Figure 2c. The elastic modulus of the M7.5 masonry infill wall was determined in accordance with the formula specified in Clause 2.2.5 of the Code for Design of Masonry Structures (GBJ 3-88) [35]. The calculation yields *E* = 1500 *f*_c_ = 2595 MPa (with *f*_c_ = 1.73 MPa), and a Poisson’s ratio of 0.2 is adopted. Furthermore, based on Clause 2.2.2 of the GBJ 3-88 [35], the design value of the axial tensile strength is taken as *f*_t_ = 0.20 MPa.

The damping ratio for both concrete and masonry walls is set to 0.05. The density of concrete and mortar is 2400 kg/m^3^, that of steel reinforcement is 7900 kg/m^3^, and that of masonry is 1800 kg/m^3^. The elastic parameters of all materials are presented in Table 2.

### 2.3. Load and Boundary Conditions

**Figure 3:**
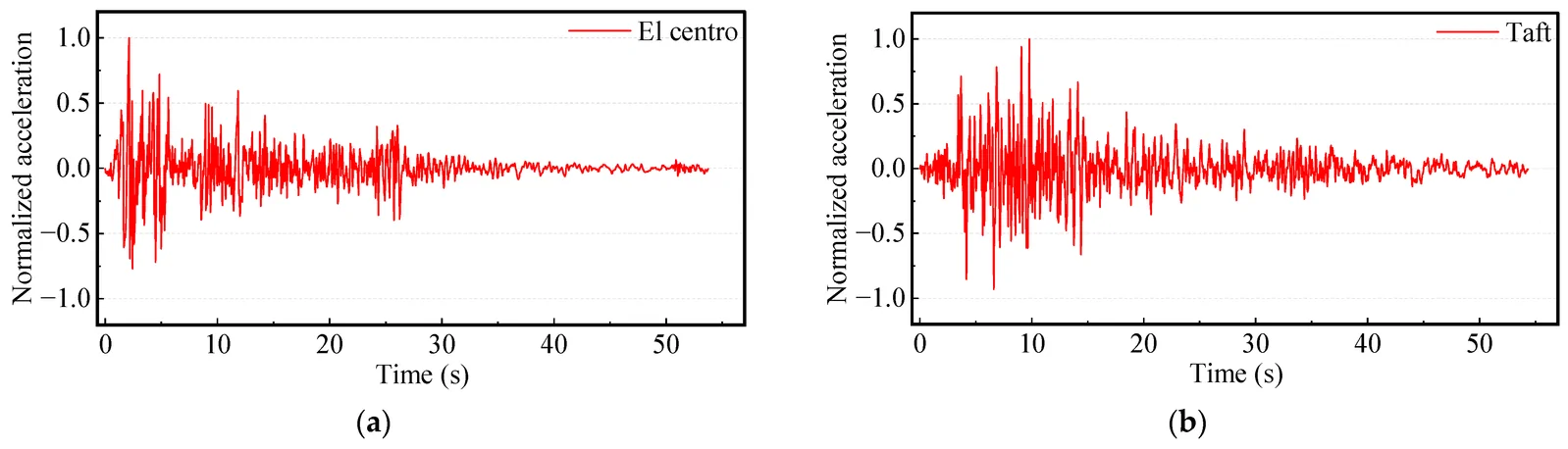
Time-history curves of ground motion records. (**a**) El centro wave; (**b**) Taft wave.

The floor dead load is specified as 5.0 kN/m^2^, and the floor live load as 2.0 kN/m^2^. For the roof, the dead load is set to 7.0 kN/m^2^, with the live load being 0.5 kN/m^2^. The loading procedure is divided into two sequential steps: first, the model’s dead and live loads are applied, followed by the seismic load, which is introduced at the model’s base in the form of acceleration. The El Centro and Taft earthquake waves are selected based on the structure’s site classification and design seismic group. For each earthquake wave, the dynamic responses of the model are computed under both the design-basis earthquake (0.2 g) and the maximum considered earthquake (0.4 g). The earthquake waves are applied at the base of the first-floor columns, with the acceleration direction aligned with the horizontal longitudinal axis. The normalized time–history curves of the earthquake waves are presented in Figure 3.

**Figure 4:**
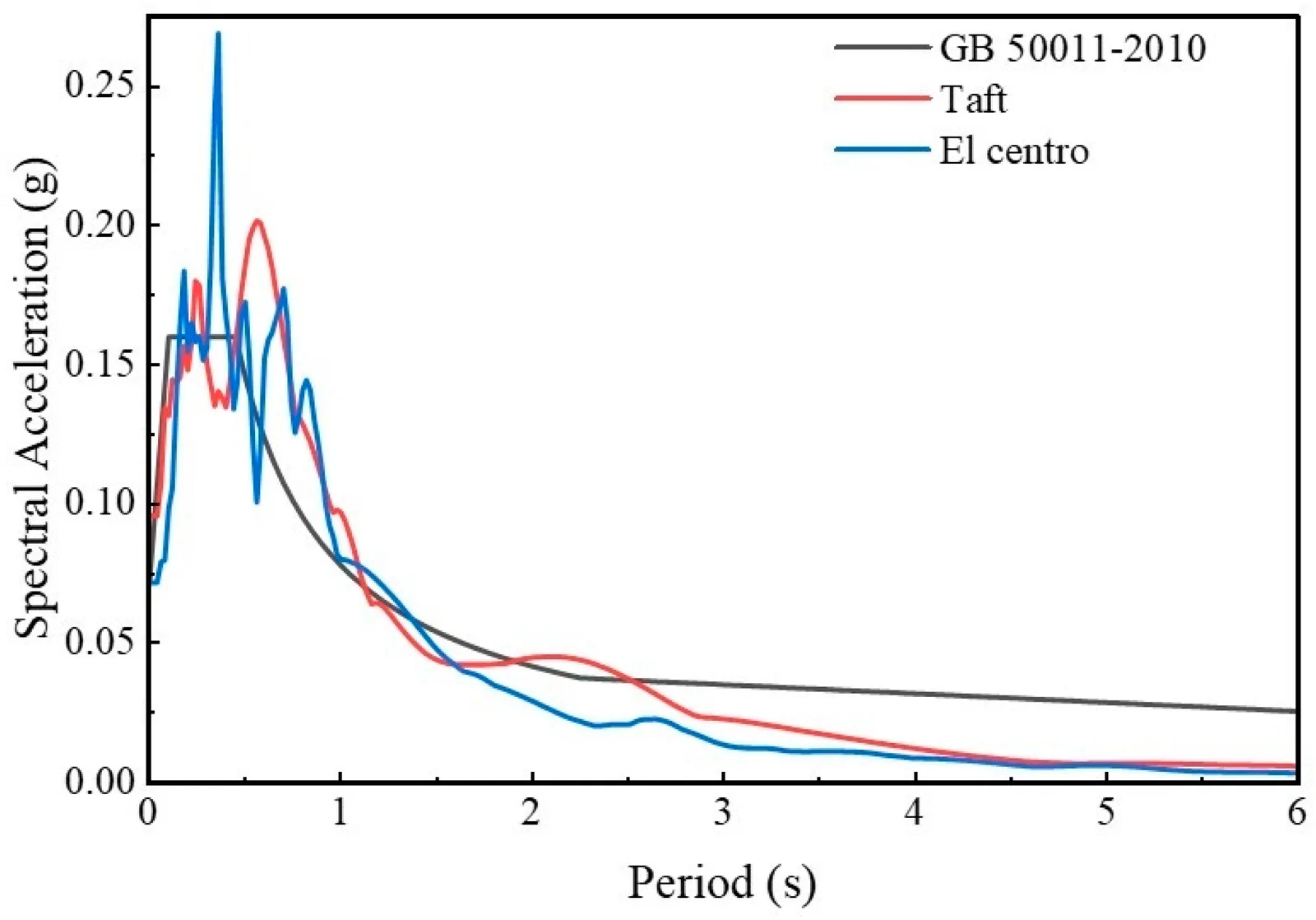
Comparison of Seismic Response Spectrum and Design Spectrum.

The response spectra of the selected ground motions were scaled and then compared with the design spectrum specified in the Chinese seismic design code (GB 50011-2010) [33]. The comparison shows that the scaled response spectra are generally compatible with the code design spectrum over the period range of interest, thereby verifying the reasonableness of the adopted ground motions. The comparison results are shown in Figure 4.

### 2.4. Contact Settings

To simplify the computational process, the contact interfaces between floor slabs and frames, as well as between masonry walls and frames, are defined as Tie constraints. This constraint enforces identical values for the three translational displacements (U1, U2 and U3) and three rotations (UR1, UR2 and UR3) at corresponding nodes across the contact surfaces.

The assumption of full adhesion implies zero relative displacement at the interface (i.e., no normal separation or tangential sliding), thereby allowing unrestricted transfer of both normal and shear forces. This idealization leads to an overestimation of the initial lateral stiffness of the masonry wall by precluding early softening and stiffness degradation associated with interfacial debonding and slip. Consequently, the infill wall and frame are modeled as acting monolithically, resulting in higher predicted initial stiffness values. Furthermore, the full adhesion assumption may overestimate the lateral load-bearing capacity and potentially alter the predicted failure mechanism. The model suppresses cracking along mortar joints and separation at the frame-infill boundaries—phenomena that often govern the primary failure modes in practice. As a result, the energy dissipation capacity of the wall may be overestimated, although the model retains the capability to simulate damage within both the frame and the infill wall.

The full adhesion assumption is considered reasonable under specific conditions, which define the applicability domain of the proposed FE methodology: (1) When the structure remains predominantly elastic and the induced interface shear stresses remain below the bond strength or frictional resistance capacity, relative slip is negligible. Under these circumstances, the constraint of perfect bonding provides a satisfactory approximation. (2) If the primary objective is to evaluate the structure’s fundamental period, initial stiffness, or global deformation patterns rather than its post-cracking nonlinear hysteretic behavior, the proposed computational approach offers enhanced efficiency while serving as an acceptable idealization.

However, the validity of this assumption diminishes when shear demands exceed the wall’s bond or frictional capacity, particularly during strong ground motions involving weak mortar or infill walls with low interfacial bond strength. In such scenarios, the model may fail to capture key phenomena such as pinching effects in hysteresis loops, reduced energy dissipation capabilities, or local out-of-plane failures of the infill wall.

### 2.5. Element Type and Mesh

Frame beams and frame columns are modeled using three-dimensional beam elements of type B31. Reinforcing bars for beams, columns, and floor slabs are embedded into the concrete matrix via the Rebar Layer functionality. For masonry infill walls, prevailing research employs two primary modeling paradigms: the monolithic model and the separated model. The monolithic model idealizes the infill wall as a homogeneous material, where bricks, mortar, and mortar joints are homogenized into a single continuous medium. Conversely, the separated model explicitly models bricks and mortar as distinct components, simulating their interaction through predefined contact surface properties. Typically, the separated model assigns distinct constitutive relations to bricks and mortar, offering advantages of higher fidelity to real structural behavior and superior simulation accuracy. However, it is associated with increased modeling complexity, a larger number of element discretizations, elevated computational cost, and a higher risk of convergence issues. The monolithic model can reasonably capture crack propagation and actual failure modes of infill walls while balancing computational efficiency, making it suitable for large-scale model analyses. Nonetheless, its accuracy is somewhat inferior to that of the separated model. To streamline the computational process, this study treats masonry infill walls as homogeneous materials. Additionally, an improved approach for simulating mortar surface layers is adopted: the Composite Layup feature in ABAQUS is utilized to laminate mortar material onto the surface of masonry infill walls.

Both floor slabs and infill walls are modeled using shell elements of type S4R. The S4R element is a four-node, reduced-integration shell element with hourglass control, capable of simulating finite membrane strains and arbitrary large rotations. In this study, the masonry infill walls are primarily subjected to in-plane loading, where the structural response is governed by membrane action and bending. Consequently, through-thickness stress components are negligible relative to the in-plane stresses. Under these conditions, the plane-stress assumption inherent in shell theory is mechanically justified. Furthermore, the wall thickness (*t* = 240 mm) is small compared with its in-plane dimensions (*L*/*t* = 3600/240 = 15, *H*/*t* = 4000/240 ≈ 16.7), satisfying the criteria for thin-to-moderately thick shells.

**Figure 5:**
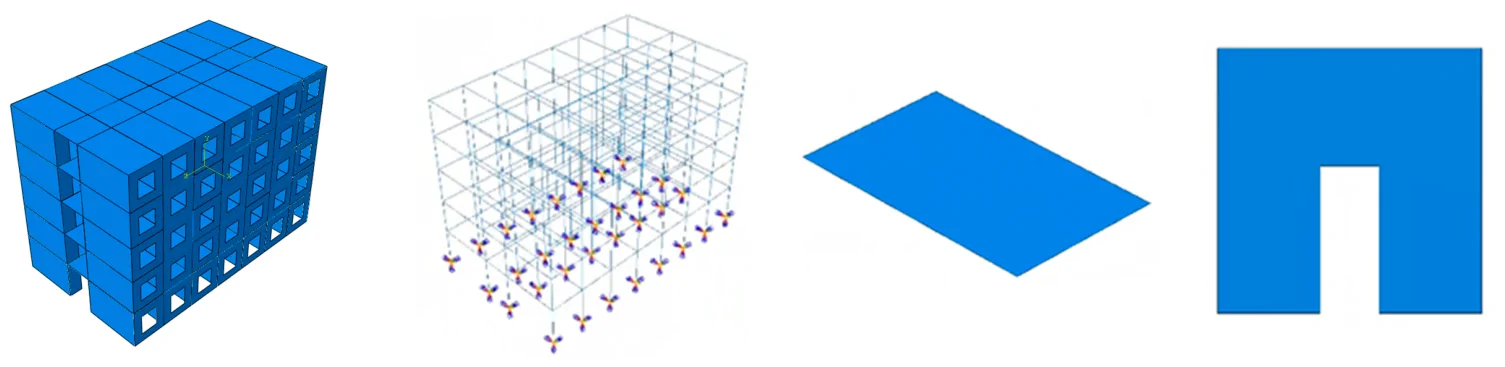
Finite element model of RC frame structure. (**a**) Overall model; (**b**) RC frame; (**c**) Floor slab; (**d**) Masonry infill wall.

The global and local finite element models of the RC frame are presented in Figure 5. Both the frame members and the floor slab are discretized with a mesh size of 500 mm, while the walls are discretized with a finer mesh size of 300 mm, as a mesh size smaller than 300 mm for the walls led to severe convergence difficulties during the analysis, while the adopted mesh sizes provide a good balance between accuracy and computational efficiency. The analyses were performed using the explicit dynamics solver in ABAQUS/Explicit, and the convergence of the solution was assessed using the default criterion in ABAQUS; the ratio of the largest residual force in the model to the average force is below 5 × 10^−3^.

### 2.6. Mechanical Validity of the Mortar Surface Layers in the Composite Layup

The reinforced masonry wall is modeled as a laminated composite layer, wherein the masonry substrate and the mortar reinforcement layer are discretized into distinct laminae, each assigned specific thicknesses, orientations, and material properties. This idealization is mechanically justified under the following conditions:

(1) Perfect Interlaminar Bonding (No Slip): Full adhesion between the reinforcement and the masonry substrate is assumed prior to failure. This assumption is consistent with the dominant failure modes observed in Ref. [36], where debonding was not the primary mechanism, and aligns with the surface preparation protocols employed in the experimental program. (2) Thin Layer Assumption: The M10 mortar reinforcement layer (t = 40 mm) satisfies the criteria that its thickness is negligible relative to both the in-plane dimensions and the radius of curvature. Consequently, the Kirchhoff–Love kinematic assumptions inherent in classical laminate theory remain valid. (3) Through Thickness Integration Accuracy: Sufficient integration points through the shell thickness ensure the accurate representation of the equivalent laminate’s axial and bending stiffnesses, thereby capturing the overall mechanical response of the strengthened wall with physical fidelity.

It is noted that this global equivalent shell approach cannot resolve localized phenomena at the micro-scale, such as brick-mortar interface debonding or stair-step crack propagation patterns.

## 3. Comparison of Numerical Analysis Methods

**Figure 6:**
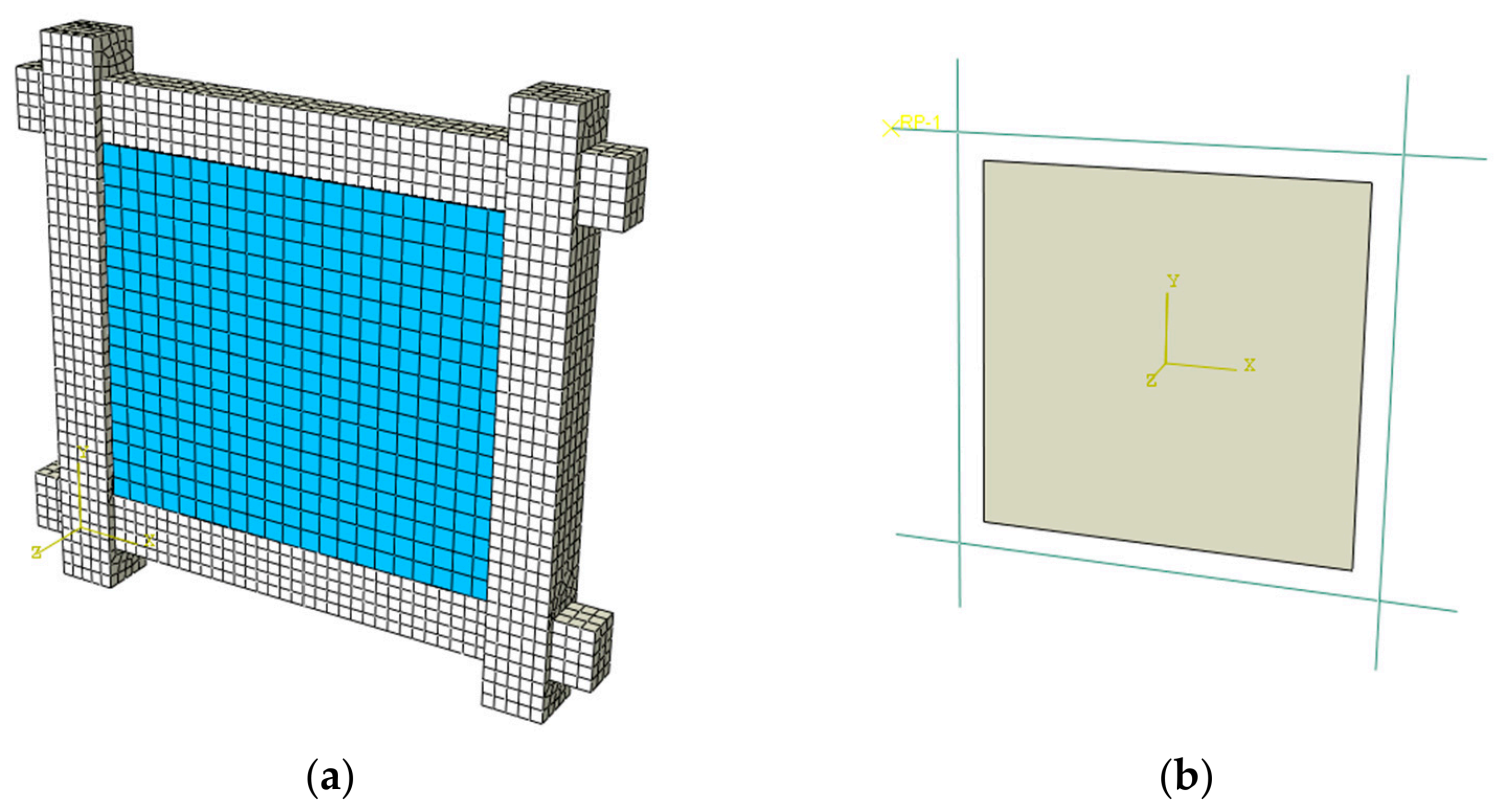
Finite element models. (**a**) 3D solid model; (**b**) simplified model.

To compare the calculation results of different simulation analysis methods, a single-story single-bay frame was selected for simulation. A 3D solid model of the frame-infilled wall system and a simplified model combining beam elements and shell elements were constructed respectively, as illustrated in Figure 6.

**Figure 7:**
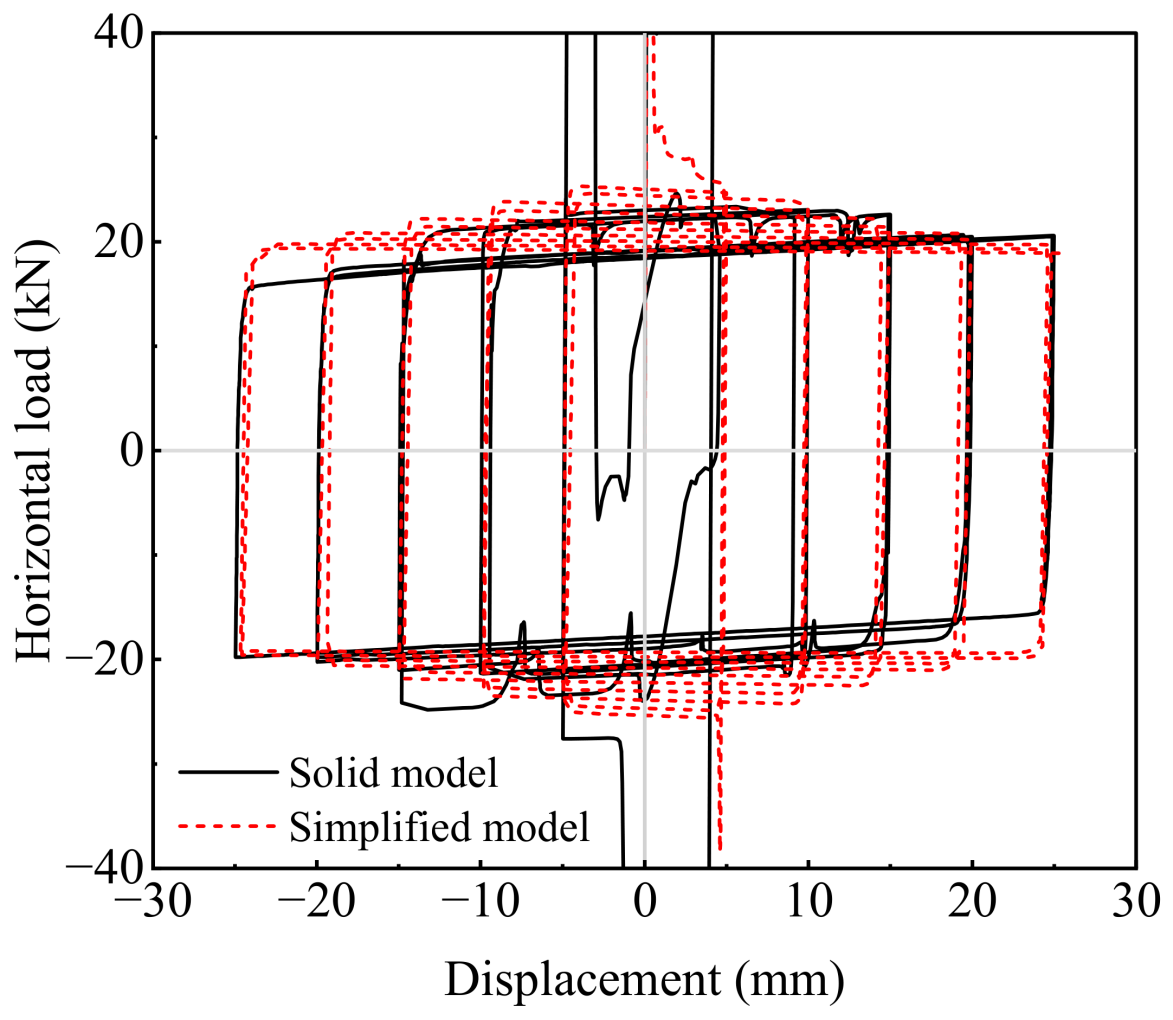
Comparison of the hysteresis curves obtained from the two models.

Horizontal cyclic loads were applied at the beam ends atop the columns of the two models, respectively, to simulate seismic actions. The validity of the numerical simulation method was verified by comparing the hysteresis curves at the load application locations. As illustrated in Figure 7, the hysteresis curves derived from the two models are in good agreement, thereby confirming the validity of the simplified model.

Although the calculated results show little difference compared with the non-simplified model, the simplified model still has its limitations. One limitation is its inability to accurately capture potential debonding phenomena at the interfaces between the masonry infill wall and the frame members, or between the mortar reinforcement layer and the wall substrate.

## 4. Numerical Analysis Cases

**Figure 8:**
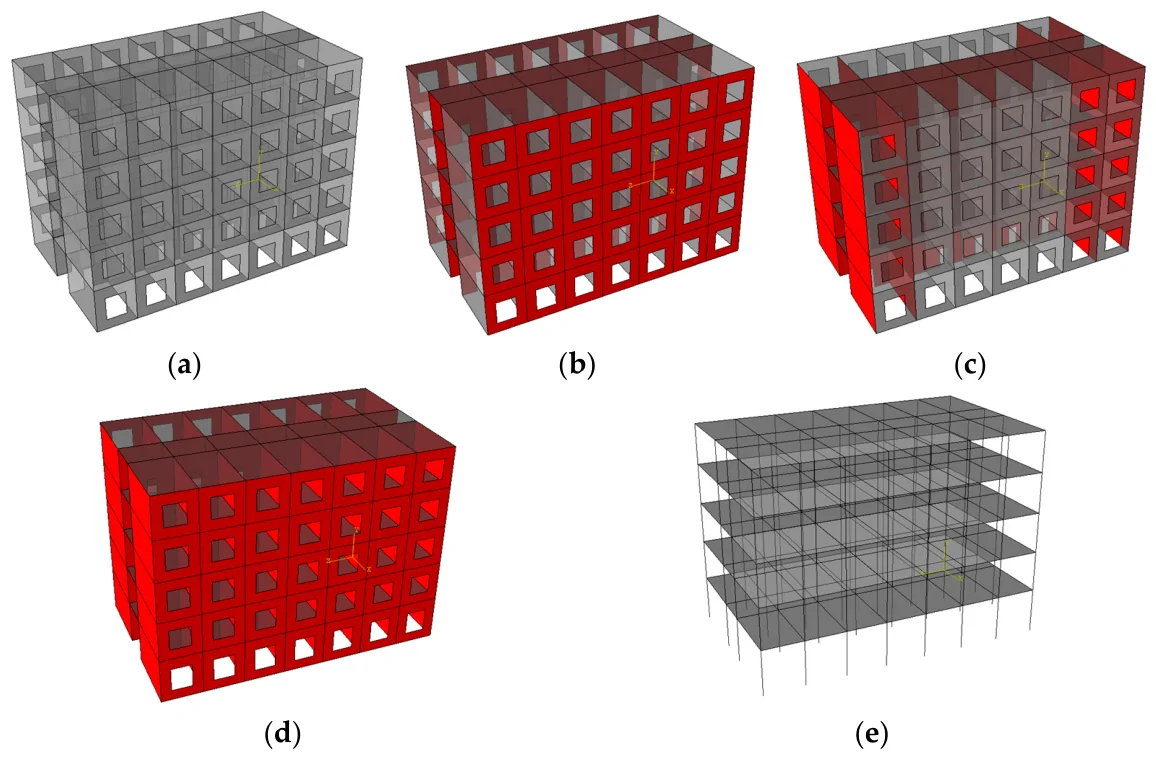
Finite element models. (**a**) MRC-1; (**b**) MRC-2; (**c**) MRC-3; (**d**) MRC-4; (**e**) RC.

To investigate the effect of masonry infill walls strengthened with mortar layers on the seismic performance of structures, five distinct computational models were established, as illustrated in Figure 8. The definition of the computational model is shown in Table 3.

For all cases, the thickness of the mortar layer is 40 mm, and the strengthening is applied to both sides of the walls.

## 5. Results Analysis

### 5.1. Natural Period of Vibration

**Figure 9:**
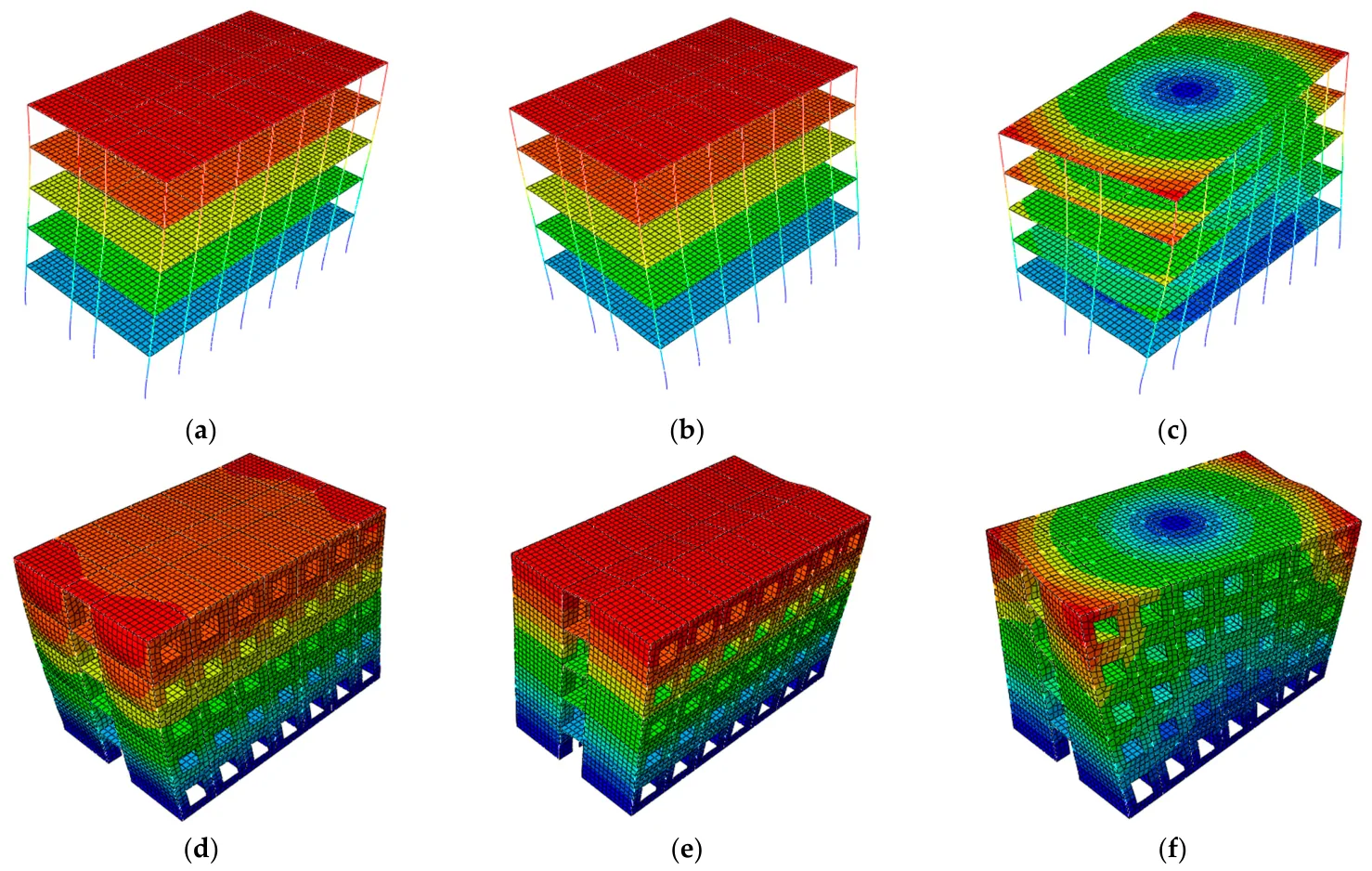
Mode shapes of models. (**a**) 1st mode shape of RC; (**b**) 2nd mode shape of RC; (**c**) 3rd mode shape of RC; (**d**) 1st mode shape of MRC-1; (**e**) 2nd mode shape of MRC-1; (**f**) 3rd mode shape of MRC-1.

Modal analysis was performed on four groups of models with distinct reinforcement configurations, and the computed results are presented in Table 4. It can be observed that different reinforcement approaches exert heterogeneous impacts on the fundamental natural periods of vibration of frame structures, with the most pronounced influence on the second mode dominated by translational motion. Mode shapes of models RC and MRC-1, shown in Figure 9.

The period ratio is defined as the ratio of the third period, which is dominated by torsional response, to the first period, which is dominated by translational response, *T*_3_/*T*_1_. The period reduction coefficient is defined as the reduction in the first period, *T*_infilled_/*T*_bare_, which quantifies the extent to which the infill walls shorten the fundamental period of the structure. The period ratios of models with infill walls are lower than those of the bare frame model RC. The period ratio of Model MRC-2 exceeds 0.9, indicating that reinforcing only vertical infill walls tends to induce torsional effects—a scenario that should be avoided in practical engineering practice. The period reduction coefficients for the four infill-wall-equipped models range from 0.39 to 0.45.

Clause 4.3.17 of the Technical Specification for Concrete Structures of High-Rise Buildings [37] specifies that the calculated natural vibration period reduction coefficient for high-rise frame structures should be taken as 0.6 to 0.7, whereas the Code for seismic design of buildings [38] does not provide explicit provisions for the value of this coefficient. The case study in this research involves a 5-story frame structure, where the lateral stiffness of infill walls is greater than that of frame columns. Additionally, multi-story frame structures may be more significantly affected by the stiffness of infill walls compared with high-rise frame structures. Thus, the period reduction coefficient in this study is smaller than the values specified in [37]. In future revisions of design codes, the determination of period reduction coefficients for multi-story frame structures should comprehensively consider factors such as the number of stories, building height, layout, and quantity of infill walls.

### 5.2. Inter-Story Drift Angle

**Figure 10:**
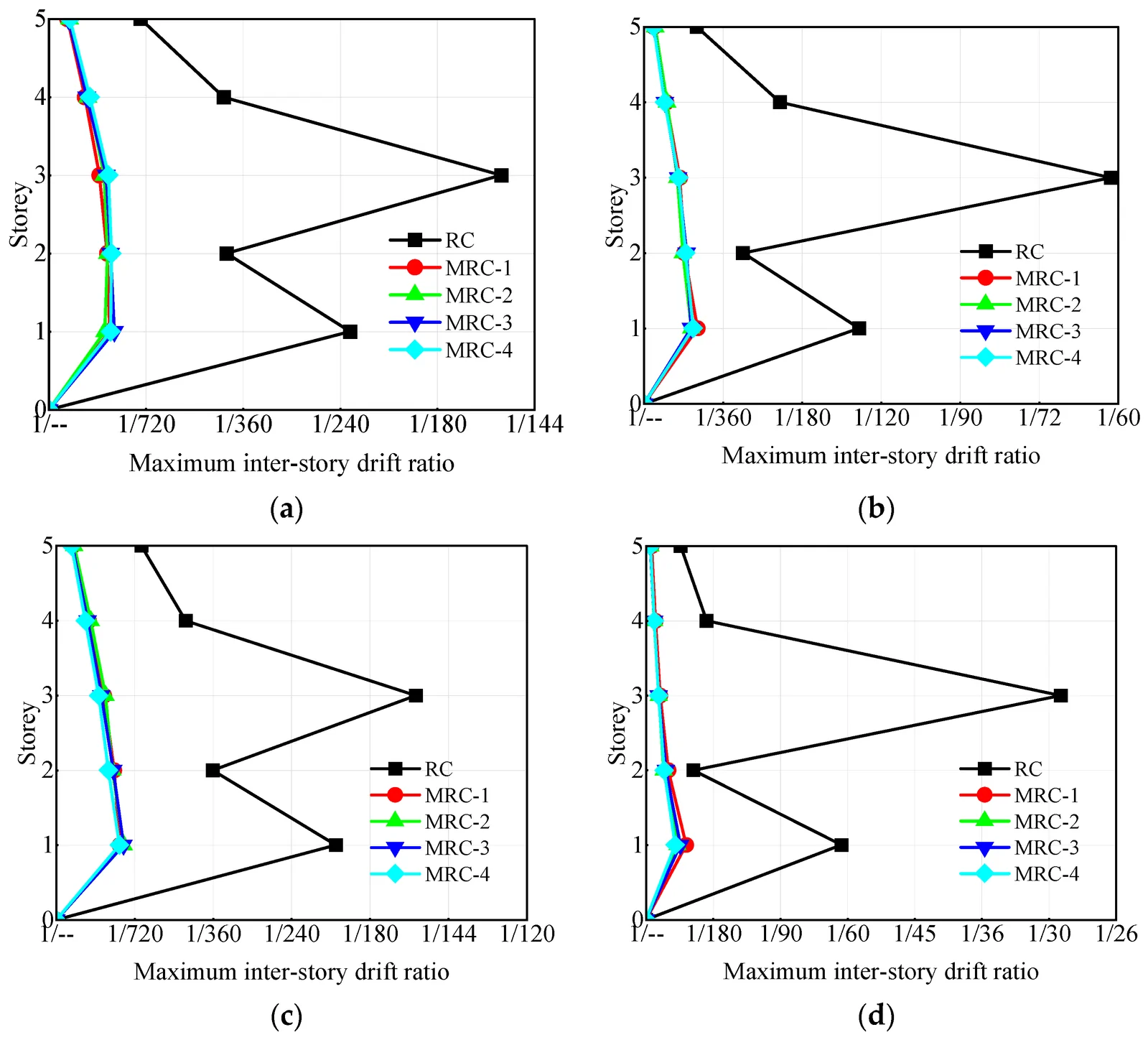
Maximum inter-story drift ratio of the floor. (**a**) El-200 gal; (**b**) El-400 gal; (**c**) Taft-200 gal; (**d**) Taft-400 gal.

This section examines the influence of the quantity and distribution of masonry walls strengthened with mortar layers on the seismic performance of RC frame structures. Time-history analyses were conducted using the El Centro and Taft earthquake ground motions, with all calculated floor displacements corresponding to the horizontal longitudinal direction. The maximum inter-story drift ratio of each floor for the bare frame structure and four structures with distinct reinforcement configurations are compared, as presented in Figure 10. It is evident that the inter-story drift ratio response of floors is mitigated following the installation of infill walls. As illustrated in Figure 10b,d, under identical seismic loading, infill walls exert the most pronounced reduction effect on the inter-story drift ratio of mid-rise floors, while their influence on upper floors is relatively weaker. In comparison to the bare frame model RC, models equipped with masonry infill walls achieve a minimum inter-story drift ratio reduction of 57.02%.

Overall, the maximum inter-story drift ratio of models MRC-3 and MRC-4 exhibit negligible differences, indicating that the strengthening effects of the full infill wall reinforcement case and the 50% infill wall reinforcement case are approximately equivalent. Among the various reinforcement schemes, model MRC-4 (with full infill wall reinforcement) demonstrates the smallest seismic response across all floors. In the current numerical analysis cases, however, from an economic perspective, the infill wall reinforcement configuration of model MRC-3 is more suitable for practical engineering applications.

### 5.3. Comparison of Seismic Forces

#### 5.3.1. Floor Shear Force

Floor shear force can reflect the inter-story shear resistance capacity of a structure. The seismic floor shear force response time histories of the structure were derived from the acceleration response time histories and structural mass of each model. The peak seismic shear forces at each floor under various longitudinal excitation conditions were extracted and plotted as the maximum seismic floor shear forces for each stage. The calculation formula for floor shear force is as follows:

(11) $$V_{i}\left(t\right)=\sum_{j=i}^{n}m_{j}a_{j}\left(t\right)$$

where *i* and *j* denote floor indices; *m_j_* represents the mass of the *j*-th floor; and *a_j_* is the centroid acceleration of the corresponding floor. The floor shear force was calculated using simultaneous signed accelerations. Specifically, at each time step, the signed acceleration responses of all floors were extracted simultaneously, and the floor shear was computed from the inertia forces obtained by multiplying the floor masses with their corresponding signed accelerations at that instant. The maximum floor shear was then identified from the resulting time history.

**Figure 11:**
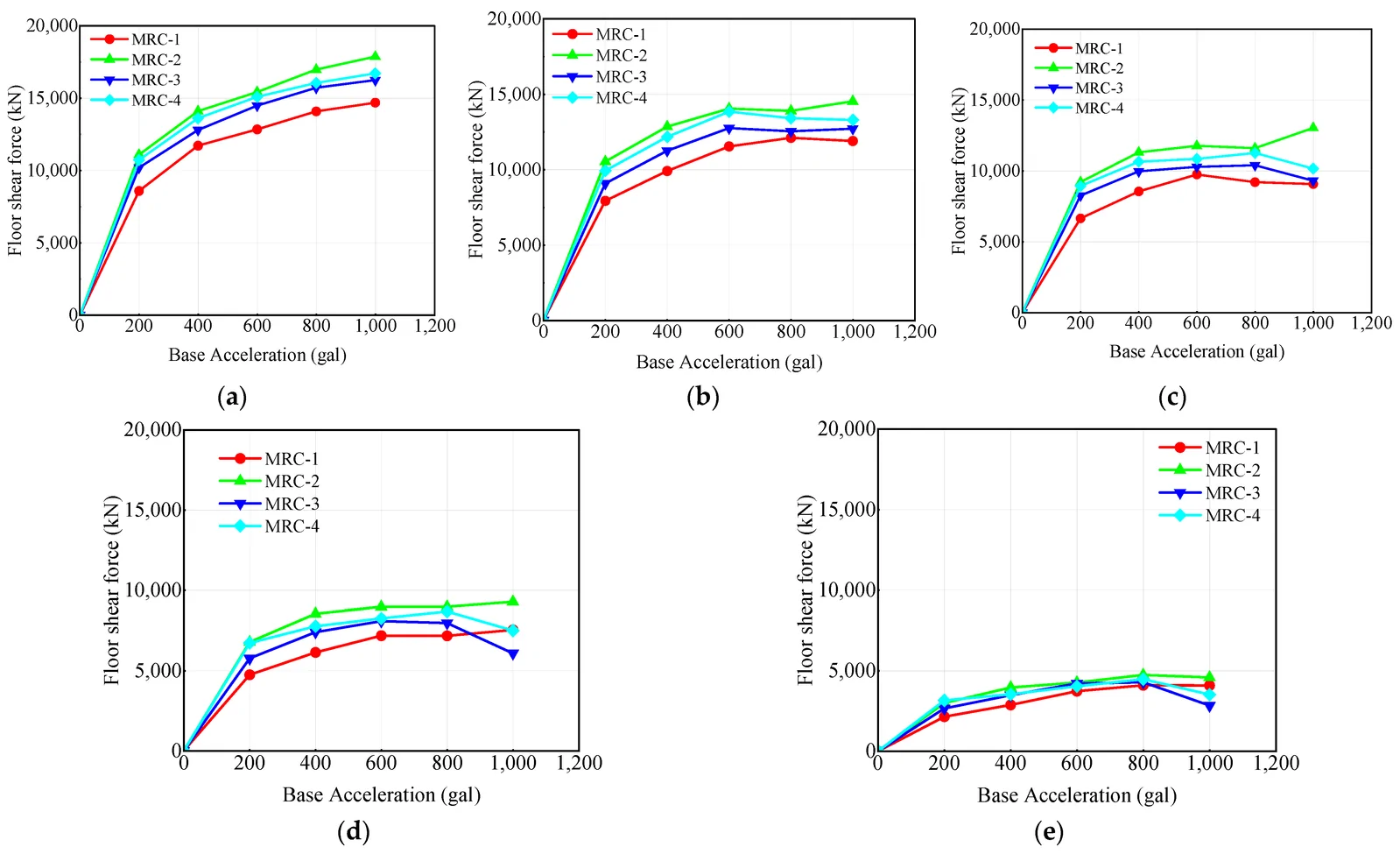
Maximum shear force on each floor. (**a**) 1st Floor; (**b**) 2nd Floor; (**c**) 3rd Floor; (**d**) 4th Floor; (**e**) 5th Floor.

The peak floor shear forces of infilled wall structures under El Centro earthquake ground motions with varying amplitudes were calculated and compared, and the results are presented in Figure 11. To accurately determine floor shear forces, additional cases with earthquake ground motion amplitudes exceeding 400 gal were included in this section.

For earthquake ground motion amplitudes below 600 gal, the magnitude of floor shear forces increases with the rise in peak base input acceleration. Notably, under different earthquake ground motions, the shear force distribution patterns of the same model differ. The following conclusions can be drawn: when the earthquake ground motion amplitude is below 600 gal, model MRC-2 exhibits the largest floor shear force, followed by models MRC-3 and MRC-4, while model MRC-1 shows the smallest. Among the various mortar-layer-strengthened masonry infill wall cases, model MRC-3, where half of the walls are reinforced in the longitudinal direction and half in the transverse direction, yields the minimum floor shear force, achieving a reduction of at least 7.8% compared with model MRC-4.

Combined with the conclusions from Section 5.2, it is evident that under the same earthquake ground motion, model MRC-3 achieves a reinforcement effect comparable to that of model MRC-4, while simultaneously reducing the seismic floor forces of the structure.

#### 5.3.2. Shear Force of Frame Columns

**Figure 12:**
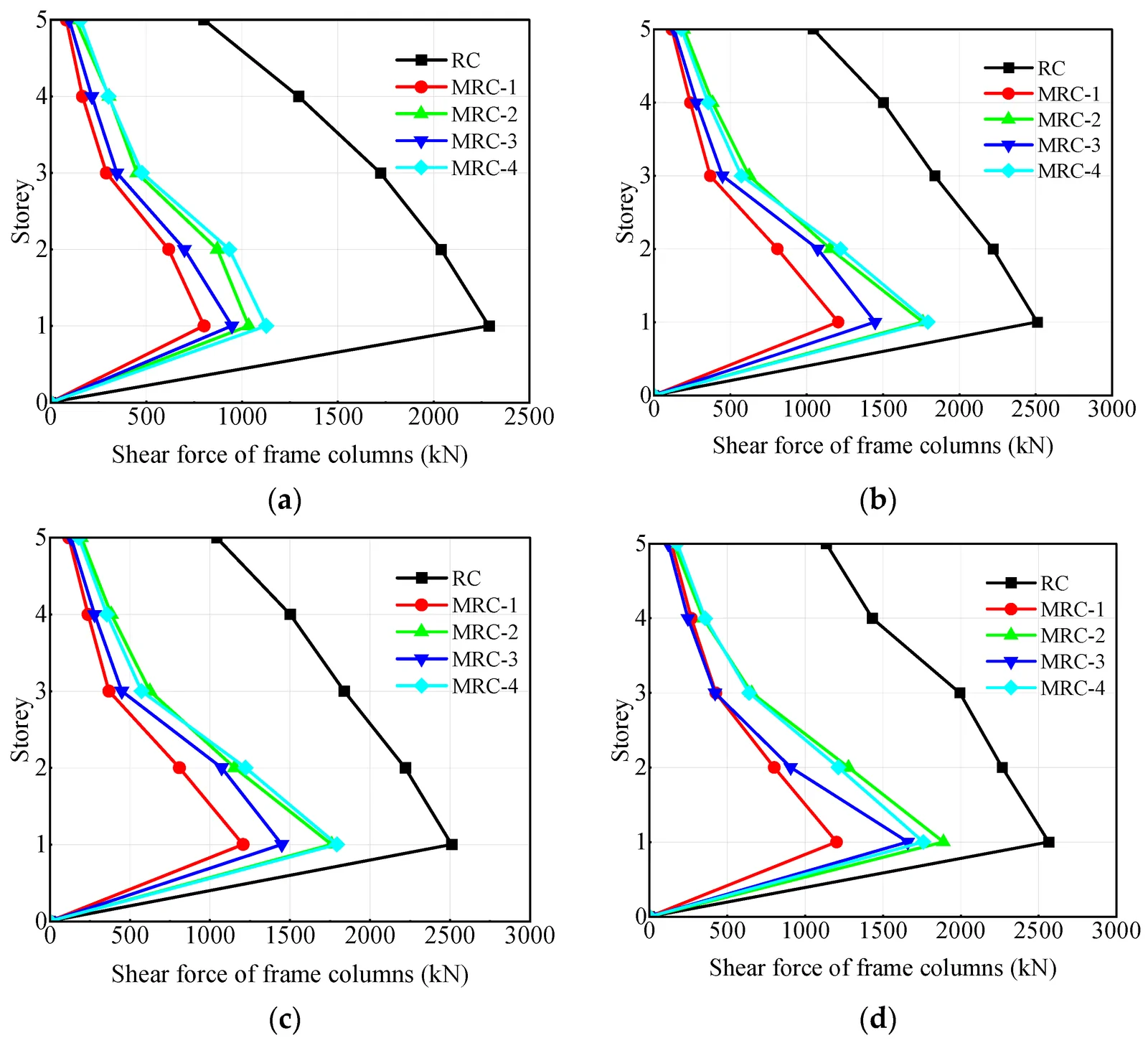
Maximum shear force of frame columns on each floor. (**a**) El-200 gal; (**b**) El-400 gal; (**c**) Taft-200 gal; (**d**) Taft-400 gal.

The comparison of shear forces in frame columns at each floor can reflect the influence of infill walls and mortar surface reinforcement on the mechanical behavior of structural members. The peak values of the sum of shear forces in frame columns at each floor under various longitudinal excitation conditions were extracted, which are defined as the maximum floor-level frame column shear forces at each stage. By comparing the floor-level frame column shear forces under seismic ground motions of different amplitudes, it is found that the addition of infill walls can reduce the shear forces in the frame columns, as illustrated in Figure 12.

Compared with model MRC-1, the models with full mortar surface reinforcement exhibit varying degrees of increase in frame column shear forces. Among these, model MRC-3 bears the minimum shear force in its frame columns; thus, from a comprehensive perspective, the reinforcement method of model MRC-3 is more favorable in terms of reducing shear demand in the frame columns. For the RC frame structure considered in this paper, it is recommended to adopt the reinforcement approach of model MRC-3 in structural retrofitting design.

### 5.4. Seismic Load-Carrying Capacity

**Figure 13:**
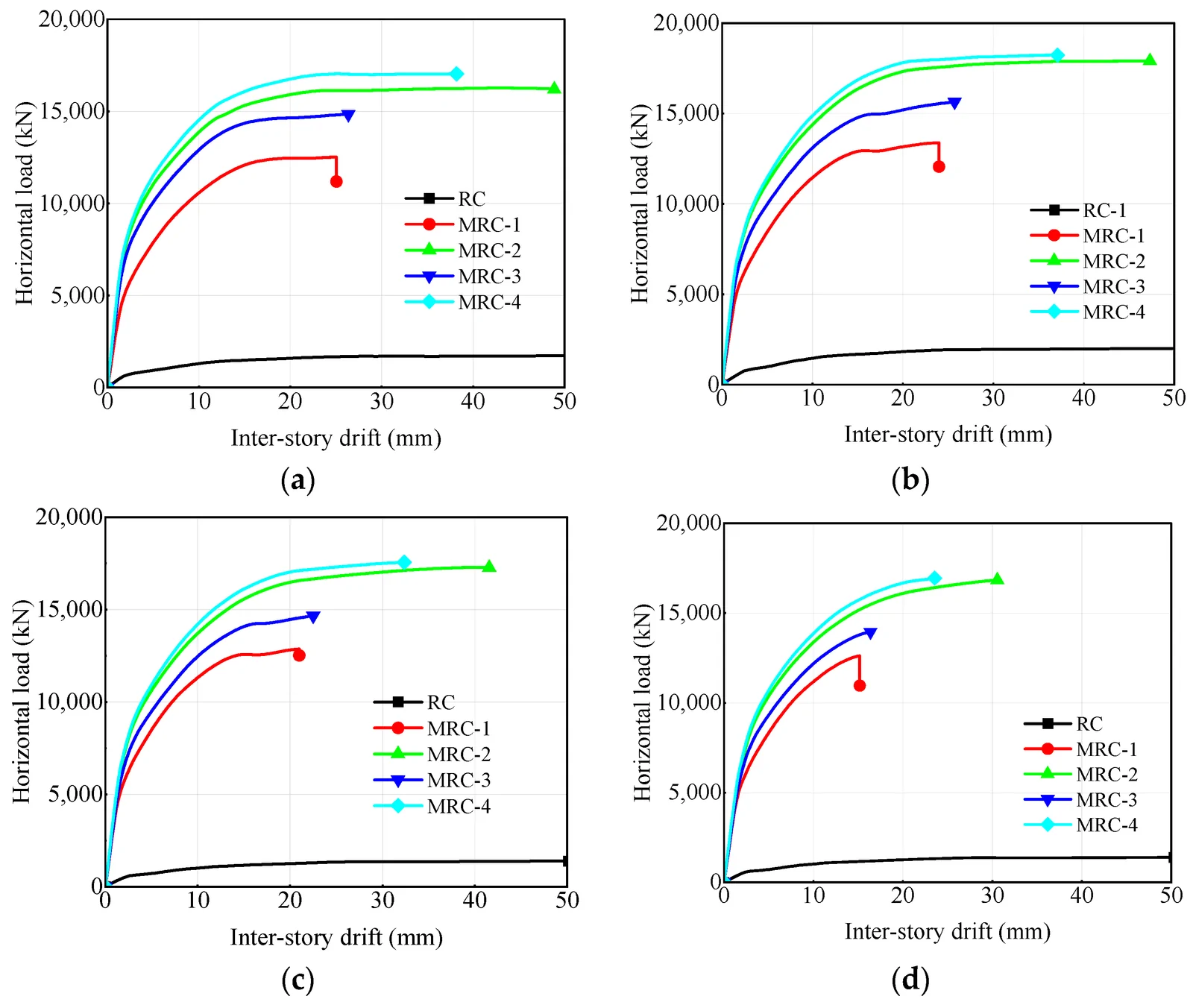
Load–displacement curves of the frame on each floor. (**a**) 1st Floor; (**b**) 2nd Floor; (**c**) 3rd Floor; (**d**) 4th Floor.

Pushover analysis, a static nonlinear analysis method, enables effective assessment of the nonlinear response characteristics of structures under seismic excitation and reveals the full-range mechanical behavior of structures from the elastic stage, through yielding, to ultimate failure. In this section, horizontal loads distributed in accordance with the first mode shape were applied to different frame models. The control node for load application is taken at the geometric centre of each story; the target displacement corresponds to a 2% inter-story drift; the increment size is 0.05% of the target displacement, and the capacity is defined as the peak of the load–displacement curve. By incrementally increasing the lateral load until the structure reached the target displacement, the horizontal load–inter-story drift curves of each model were obtained, as depicted in Figure 13. Owing to model convergence issues during the calculation process, the results for some models only cover data up to the peak load. Analyses terminated by non-convergence are therefore reported separately as “numerically terminated”, and only results up to the last converged step are used for the quantitative capacity definition.

It can be observed that the seismic load-carrying capacity of the strengthened models (MRC-2, MRC-3, and MRC-4) was increased by at least 7.2 times compared with the bare RC frame model, and by 18.57% to 36.19% compared with the unreinforced model MRC-1. Among the three strengthened models, model MRC-4 sustained the largest horizontal load at the same inter-story drift, indicating it has the highest seismic load-carrying capacity. The seismic load-carrying capacities of models MRC-2 and MRC-4 were not significantly different, which suggests that the reinforcement of lateral walls has a limited impact on the lateral load-carrying capacity.

### 5.5. Comparison of Plastic Strain

**Figure 14:**
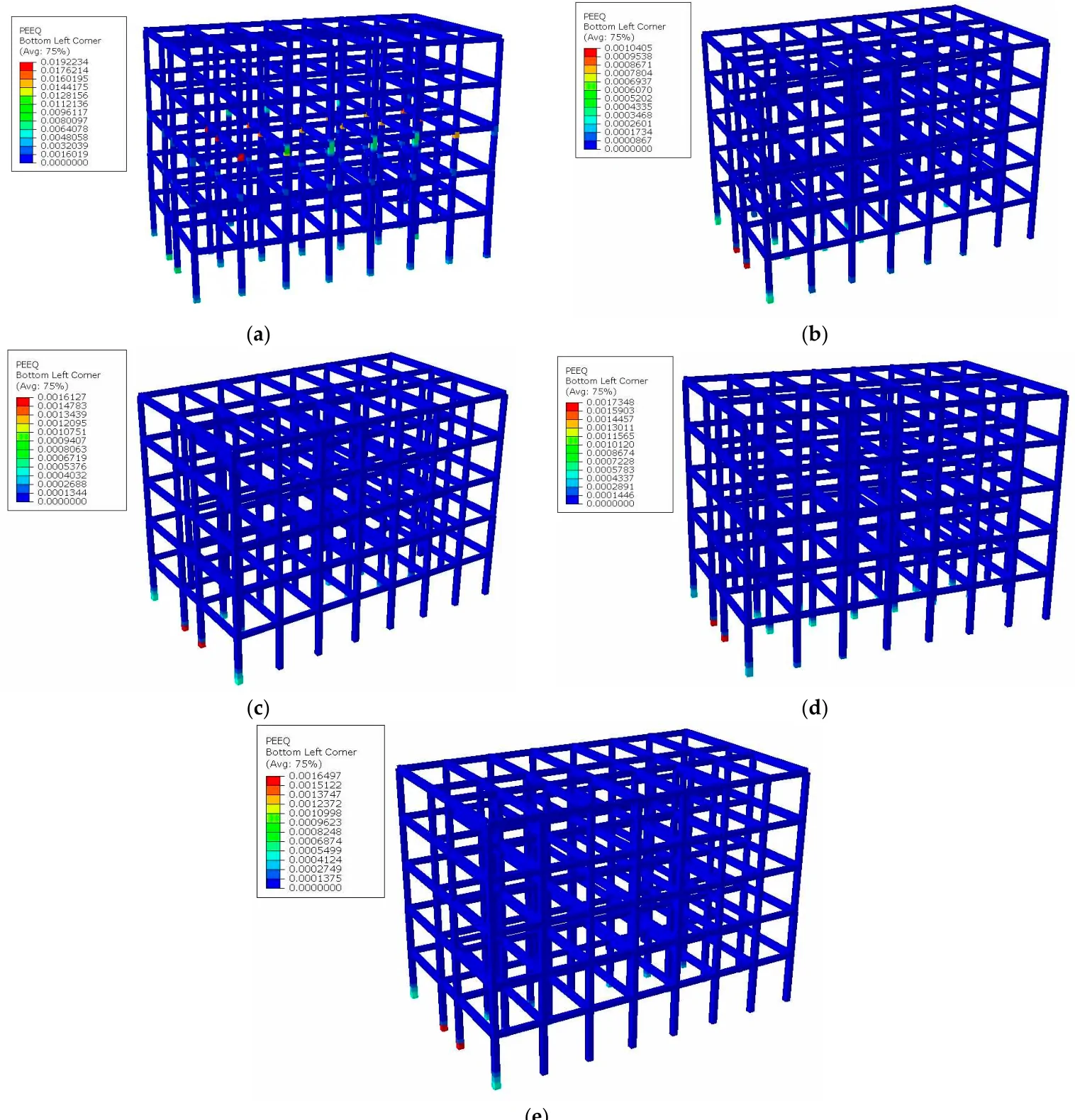
Plastic strain distribution of the model. (**a**) RC; (**b**) MRC-1; (**c**) MRC-2; (**d**) MRC-3; (**e**) MRC-4.

The plastic strain contours for all numerical examples under the El Centro wave (400 gal) are presented in Figure 14. A bare RC frame, devoid of masonry infills, serves as the control case for this comparison. It is observed that plastic strain in the bare frame concentrates at the tops of the third-story columns; see Figure 14a. In contrast, for the infill wall case, plastic strain is predominantly localized at the bases of the first-story columns, with a maximum value lower than that of the bare frame.

## 6. Conclusions

This study performed an elastoplastic dynamic time–history analysis on a five-story reinforced concrete (RC) frame structure, focusing on structural displacement and floor shear forces to investigate the effect of mortar-surfaced masonry infill wall reinforcement on the seismic performance of RC frame structures. The key conclusions are as follows:

(1) The inter-story drift response of floors was significantly reduced after infill walls were installed. Compared with the bare RC frame model, models with masonry infill walls achieved an inter-story drift reduction of at least 57.02%. Overall, the inter-story drift responses of models with fully reinforced infill walls were roughly consistent with those of models where 50% of the walls were reinforced in the transverse and longitudinal directions, respectively. Thus, reinforcing 50% of the masonry infill walls in both transverse and longitudinal directions is more suitable for practical engineering applications.
(2) A comparison of model calculation results revealed that the addition of infill walls significantly reduced the floor-level frame column shear forces of the structure. Among the infill wall-reinforced models, model MRC-2 exhibited the largest floor shear forces, while models MRC-3 and MRC-4 showed smaller values. This indicates that among different infill wall reinforcement schemes, the scheme of reinforcing 50% of the walls in the vertical and horizontal directions, respectively, resulted in the smallest floor shear forces, achieving a reduction of at least 7.8% compared with the scheme of fully reinforcing the walls. Model MRC-3 achieved a reinforcement effect comparable to that of model MRC-4, while simultaneously reducing the structural seismic floor forces and the shear forces borne by the frame columns. In the current numerical analysis cases, from a comprehensive perspective, the reinforcement method of model MRC-3 is more suitable for frame column strengthening, and it is recommended to adopt partial infill wall reinforcement in structural retrofitting design.
(3) Pushover analysis results showed that the seismic load-carrying capacity of models MRC-2, MRC-3, and MRC-4 was increased by at least 7.2 times compared with the bare RC frame model, and by 18.57–36.19% compared with the unreinforced model MRC-1. Thus, mortar-surfaced infill wall reinforcement can effectively improve the seismic load-carrying capacity of frame structures. The seismic load-carrying capacities of models MRC-2 and MRC-4 were not significantly different, indicating that the reinforcement of transverse walls has a limited impact on lateral load-carrying capacity.
(4) Although the calculated results show little difference compared with the non-simplified model, the simplified model still has its limitations. The constraints of the proposed finite element simulation methodology are explicitly stated, particularly its inability to accurately capture potential debonding phenomena at the interfaces between the masonry infill wall and the frame members, or between the mortar reinforcement layer and the wall substrate.

Notably, all quantitative conclusions rely on an idealized interface model and cannot serve as definitive predictions of actual seismic performance due to methodological limitations. Thus, experimental testing is essential to quantify the reinforcement effects in practical applications. It is also important to note that this study was limited to a single geometric configuration of the frame structure with one specific mortar layer thickness, one type of masonry wall strengthened by mortar, two natural ground motion records, and an experimentally unverified FE method. Consequently, the conclusions drawn herein are restricted to the scope of this specific case study. Further extensive experimental and numerical investigations are required to generalize these findings.

## Figures and Tables

**Table 1:** Reinforcement of frame columns.

	1st Floor	2nd Floor	3rd Floor	4th Floor	5th Floor
Corner column	8Φ16 ^1^	8Φ16	8Φ14	8Φ14	8Φ14
Edge column	8Φ14	8Φ14	8Φ12	8Φ12	8Φ12
Column stirrups	Φ8@150/400 ^2^				

^1^ The symbol Φ denotes Grade I plain round reinforcing bar. ^2^ Φ8@150/400 denotes 8 mm stirrups spaced at 150 mm within confinement regions and 400 mm outside these regions.

**Table 2:** Elasticity parameters of materials.

	Elastic Modulus/MPa	Poisson’s Ratio
C20 concrete	25,500	0.2
HPB235 steel reinforcement	210,000	0.3
M7.5 infill wall	2595	0.2
M10 mortar	7313	0.2

**Table 3:** The definition of the computational model.

Model	Definition
MRC-1	All infill walls are unreinforced
MRC-2	All longitudinal infill walls are strengthened, while transverse infill walls remain unreinforced
MRC-3	50% of the infill walls in both the transverse and longitudinal directions are strengthened
MRC-4	All infill walls are strengthened
RC	A bare frame model without infill walls, serving as a comparative case

**Table 4:** The natural vibration period and period ratio of the models.

	1st Period (s)	2nd Period (s)	3rd Period (s)	Period Ratio	Period Reduction Coefficient
RC	0.610	0.583	0.530	0.870	—
MRC-1	0.271	0.229	0.220	0.812	0.45
MRC-2	0.236	0.235	0.217	0.919	0.39
MRC-3	0.248	0.223	0.210	0.845	0.41
MRC-4	0.244	0.216	0.204	0.836	0.40

## Data Availability

The original contributions presented in this study are included in the article. Further inquiries can be directed to the corresponding author.

## Abbreviations

The following abbreviations are used in this manuscript:

RC	Reinforced concrete
MRC	Reinforced concrete structures with masonry infill walls
CDP	Concrete damaged plasticity
